# Risk factors for postoperative deep infection in bone tumors

**DOI:** 10.1371/journal.pone.0187438

**Published:** 2017-11-09

**Authors:** Shinji Miwa, Toshiharu Shirai, Norio Yamamoto, Katsuhiro Hayashi, Akihiko Takeuchi, Kaoru Tada, Yoshitomo Kajino, Hiroyuki Inatani, Takashi Higuchi, Kensaku Abe, Yuta Taniguchi, Hiroyuki Tsuchiya

**Affiliations:** 1 Department of Orthopaedic Surgery, Kanazawa University School of Medicine, Kanazawa, Japan; 2 Department of Orthopaedics, Graduate School of Medical Science, Kyoto Prefectural University of Medicine, Kyoto, Japan; Colorado State University, UNITED STATES

## Abstract

**Background:**

Postoperative deep infection after bone tumor surgery remains a serious complication. Although there are numerous reports about risk factors for postoperative deep infection in general surgery, there is only a small number of reports about those for bone tumor surgery. This retrospective study aimed to identify risk factors for postoperative deep infection after bone tumor resection.

**Methods:**

We reviewed data of 681 patients (844 bone tumors) who underwent surgery. Associations between variables, including age, recurrent tumor, pathological fracture, surgical site (pelvis/other), chemotherapy, biological reconstruction, augmentation of artificial bone or bone cement, the use of an implant, intraoperative blood loss, operative time, additional surgery for complications, and postoperative deep infection were evaluated.

**Results:**

The rate of postoperative deep infection was 3.2% (27/844 tumors). A pelvic tumor (odds ratio [OR]: 3.4, 95% confidence interval [CI]: 1.0–11.3) and use of an implant (OR: 9.3, 95% CI: 1.9–45.5) were associated with an increased risk of deep infection.

**Conclusions:**

This retrospective study showed that pelvic tumor and use of an implant were independent risk factors for deep infection. This information will help surgeons prepare an adequate surgical plan for patients with bone tumors.

## Introduction

Deep infection is one of the most serious complications after surgery. Postoperative deep infection usually requires additional surgery, the prolonged use of antibiotics, and delays in scheduled treatment such as chemotherapy. Among orthopaedic surgeries, 9–36% of patients had deep infection after bone tumor surgery [1–5]. McDonald et al. reported that 11.8% of patients who received limb salvage surgery experienced infection, and 22.2% of patients with postoperative infection underwent amputation [6]. To improve the outcome of bone tumor surgery, the risk of each surgery should be assessed and patients with high risk of deep infection should be treated with preventive measures such as nutritional optimization, perioperative antibiotics or MRSA nasal screening and decolonization. Although there are numerous reports describing risk factors for postoperative deep infection after orthopedic surgery, including arthroplasty and spine surgery, only a small number of studies have reported those after bone tumor surgery [5,7–11]. In the reports, African-American race, local infection at the limb sparing surgery, lower WBC, BMI, age, total number of procedures, preexisting implants, infection at another site, malignant disease, hip region affected, and duration of the procedure, were independent risk factor for deep infection after bone tumor surgery [5,7,9,11]. To choose adequate surgical treatment, it is important to assess the risk of postoperative deep infection in each patient preoperatively. In the present study, the frequency of postoperative deep infection and correlations of deep infection with various clinical parameters, including age, the tumor location, the use of an implant, chemotherapy, the use of artificial bone or cement, operative time, intraoperative blood loss, biological reconstruction, and additional surgery for complications, were investigated to identify risk factors for postoperative deep infection.

## Methods

### Patients

This was a single-center, retrospective case study. Overall, 681 patients with 844 bone tumors, who underwent surgery between January 1995 and December 2015, were enrolled in this study. Metastatic bone tumors were excluded from this study. The study patients comprised 390 men and 291 women whose ages ranged from 1 to 92 years (mean age, 28.0 years). The tumor diagnoses were confirmed through histopathological examinations (Table 1). Bone tumors located in the femur (n = 273), tibia (n = 176), humerus (n = 95), pelvis (n = 80), foot (n = 64), hand (n = 58), fibula (n = 33), rib (n = 17), scapula (n = 14), ulna (n = 14), radius (n = 14), clavicle (n = 4), sternum (n = 1), and patella (n = 1) were included in this study (Table 2). Patients with spine tumors and those who underwent surgery using implants with an antimicrobial coating were excluded from this study. The reconstructions after resection of bone tumors were classified into: no reconstruction, biological reconstruction, implant replacement, use of artificial bone or cement, or composite use of the materials. This retrospective study was approved by the ethics committee of Kanazawa University. All data were fully anonymized before access by the researchers, and the ethics committee waived the requirement for informed consent.

**Table 1:** Diagnoses of the lesions.

Benign tumor		Malignant tumor	
Diagnosis	Number of lesions	Diagnosis	Number of lesions
Osteochondroma	195	Osteosarcoma	102
Bone cyst	126	Chondrosarcoma	55
GCT	83	MFH/UPS	20
Enchondroma	70	Ewing sarcoma	13
Fibrous dysplasia	27	Hemangiopericytoma	2
Chondroblastoma	20	Adamantinoma	2
NOF	20	Fibrosarcoma	1
ABC	18	Malignant GCT	1
Osteoid osteoma	14		
LCH	9		
OFD	9		
Ganglion	8		
Fibroma	5		
Chondroma	4		
BPOP	3		
Lipoma	3		
Hemangioma	2		
Others	32		

MFH, malignant fibrous histiocytoma; UPS, undifferentiated pleomorphic sarcoma; GCT, giant cell tumor; NOF, non-ossifying fibroma; ABC, aneurysmal bone cyst; OFD, osteofibrous dysplasia; LCH, Langerhans cell histiocytosis; BPOP, bizarre parosteal osteochondromatous proliferation

**Table 2:** Locations and incidence of postoperative deep infection.

Locations	Number of tumors	Infection (%)
Femur	273	5 (1.8%)
Tibia	176	13 (7.4%)
Humerus	95	1 (1.1%)
Pelvis	80	7 (8.8%)
Foot	64	1 (1.6%)
Hand	58	0 (0%)
Fibula	33	0 (0%)
Rib	17	0 (0%)
Scapula	14	0 (0%)
Ulna	14	0 (0%)
Radius	14	0 (0%)
Clavicle	4	0 (0%)
Sternum	1	0 (0%)
Patella	1	0 (0%)
Total	844	27 (3.2%)

### Outcome measure

In this study, the incidence of postoperative deep infection and its association with various factors were evaluated. The optimal cutoff levels for age, the operative time, and intraoperative blood loss were identified in receiver operator characteristic curve analysis. The patient-related parameters were as follows: age (<20 or ≥20 years), location of the tumor (pelvis or other), recurrent tumor (yes/no), pathological fracture (yes/no), and chemotherapy (yes/no). The surgery-related parameters were as follows: the use of an implant (yes/no), biological reconstruction (yes/no), use of artificial bone or bone cement for bone defect (yes/no), additional surgery for complications (yes/no), operative time (<5 or ≥5 hours), and intraoperative blood loss (<150 or ≥150 mL). Biological reconstruction was defined as the use of allograft, iliac bone, fibular bone, tibial bone, and tumor-bearing bone treated by freezing or autoclaving, for bone defect after bone tumor resection [12]. Artificial bone was defined as α-tricalcium phosphate (TCP) or β-TCP. Bone cement was defined as polymethylmethacrylate. Additional surgeries for complications were defined as surgical treatment for delayed bone union, fracture, wound dehiscence, breakage of implants, hematoma, and intestinal perforation. Postoperative deep infections were defined using the US Centers for Disease Control classifications for surgical site infections [13].

### Statistical analysis

To identify the risk factors for postoperative deep infection after bone tumor surgeries, univariate analysis was performed using the Fisher exact test. Multiple logistic regression analysis was used to identify the independent risk factors for postoperative deep infection. The parameters with univariate p values <0.1 were considered as candidates for the multiple logistic regression model. Statistical significance was defined as p < 0.05, and all analyses were performed using statistical software (EZR, Saitama Medical Center, Jichi Medical University).

## Results

### Risk factors for postoperative deep infection

Among the study patients, the incidence of postoperative deep infection was 3.2% (27/844 operations). Results of univariate analyses showed that a pelvic tumor, chemotherapy, the use of an implant, biological reconstruction, additional surgery for complications, operative time ≥5 hours, and intraoperative blood loss ≥150 mL were significantly associated with an increased risk of postoperative deep infection (Tables 3 and 4). The use of artificial bone or cement was significantly associated with a decreased risk of postoperative deep infection.

**Table 3:** Results of univariate analysis of the patient-related parameters.

Factor		Number (%) of tumors with deep infection	OR	95% CI	p value
Age	≥20 years	18/429 (4.2%)	2.23	0.91–5.99	0.072
	<20 years	8/415 (1.9%)			
Tumor location	Pelvis	7/80 (8.8%)	3.75	1.29–9.71	0.008
	Other	19/764 (2.5%)			
Recurrent tumor	Yes	2/95 (2.1%)	0.65	0.07–2.69	0.758
	No	24/749 (3.2%)			
Pathological fracture	Yes	0/37 (0%)	0.00	0.00–3.38	0.623
	No	26/807 (3.2%)			
Chemotherapy	Yes	16/108 (14.8%)	12.55	5.18–31.94	< 0.001
	No	10/736 (1.4%)			

OR, odds ratio; CI, confidence interval.

The p values were calculated with Fisher exact test.

**Table 4:** Results of univariate analysis of the surgery-related parameters.

Factor		Number (%) of tumors with deep infection	OR	95% CI	p value
Use of an implant	Yes	22/130 (16.9%)	35.89	11.88–145.94	< 0.001
	No	4/714 (0.6%)			
Biological reconstruction	Yes	21/134 (15.7%)	26.02	9.31–90.21	< 0.001
	No	5/710 (0.7%)			
Artificial bone or cement	Yes	2/305 (0.7%)	0.14	0.02–0.58	0.001
	No	24/539 (4.5%)			
Additional surgery for complications	Yes	8/36 (22.2%)	12.43	4.30–33.32	< 0.001
	No	18/808 (2.2%)			
Operative time	≥5 h	18/84 (21.4%)	25.39	10.06–70.29	< 0.001
	<5 h	8/760 (1.1%)			
Intraoperative blood loss	≥150 mL	21/259 (8.1%)	10.20	3.69–35.06	< 0.001
	<150 mL	5/585 (0.9%)			

OR, odds ratio; CI, confidence interval.

The p values were calculated with Fisher exact test.

Multiple logistic regression analysis included the following 8 variables: pelvic tumor, chemotherapy, the use of an implant, biological reconstruction, augmentation of artificial bone or cement, additional surgery for complications, operative time ≥5 hours, and intraoperative blood loss ≥150 mL. Result of multivariate analysis showed that pelvic tumor and use of an implant were significantly associated with an increased risk of postoperative infection (Table 5).

**Table 5:** Risk factors for postoperative deep infection according to multivariate analysis.

Factor	OR	95% CI	p value
Pelvic tumor	3.42	1.04–11.30	0.044
Operative time ≥5 h	2.17	0.63–7.49	0.221
Use of an implant	9.28	1.89–45.50	0.006
Biological reconstruction	4.20	0.96–18.30	0.057
Chemotherapy	2.18	0.74–6.42	0.156
Additional surgery for complications	1.57	0.53–4.63	0.412
Artificial bone or cement	1.65	0.25–10.80	0.603
Intraoperative blood loss ≥ 150 mL	0.76	0.20–2.96	0.693

OR, odds ratio; CI, confidence interval.

Values were calculated by multiple logistic regression analysis.

## Discussion

The treatment of bone tumors includes surgery, chemotherapy, radiation therapy, medications, and immunotherapy [14,15]. Surgical treatment for bone tumors comprises tumor resection and reconstruction for bone defects using an endoprosthesis, allograft, autograft, and artificial bone graft. The surgical outcomes of bone tumors such as limb function, recurrence, and complications can be influenced by several factors, including age, tumor histology (primary or metastatic tumor), chemotherapy, radiation therapy, and the surgical site. Among the complications, postoperative deep infection remains a common and severe complication after bone tumor surgery. The causes of the postoperative deep infection after tumor resection include immunocompromised patients with cancer, malnutrition, large bone and soft tissue defects, a long operative time, frequent red blood cell and platelet transfusions, neutropenia from postoperative chemotherapy, and frequent bacteremia from the use of indwelling central venous catheters [16]. The rate of postoperative deep infection after resection of bone tumor has been reported to range from 0.9% to 36% [3,4,17–20]. Postoperative deep infection requires additional treatment such as irrigation surgery, the use of antibiotics for a long period, and delays in the treatment course, which increases mortality. To improve the outcomes of patients with bone tumors, physicians need to recognize the risk factors for postoperative deep infection to determine the adequate surgical procedure.

Dietz et al. reported that 58% of orthopedic surgeries had bacterial contamination [21]. According to an intraoperative experiment, surgical wound, local bone harvested from surgical sites, surgeon’s gloves, and implants were contaminated by the same bacteria that were cultured from the room air of the operating room, and the degree of contamination increased proportionally with the exposure time [22]. The National Nosocomial Infections Surveillance (NNIS) has identified an operative time of ≥4 hours as being predictive of deep infection after general surgery procedures [23].

The associations between postoperative deep infection and chemotherapy, and radiation therapy are controversial. Leukocytopenia and neutropenia due to chemotherapy and tissue damage caused by radiation therapy are thought to be associated with postoperative deep infection. However, a study about the risk factors of postoperative infection showed that adjuvant chemotherapy and radiotherapy were not significant risk factors for infection [11]. On the other hand, a study on spinal metastases showed that radiation therapy was an independent risk factor for postoperative infection [24].

Our present study revealed that pelvic tumors significantly increased the risk of postoperative deep infection. Deep infection is one of the most frequent postoperative complications after pelvic surgery (range, 20–36%), and it requires surgical debridement and irrigation [1,3,17,20,25]. Bone tumors of the pelvis are often large, because they are diagnosed late. Furthermore, choosing an adequate surgical treatment is particularly difficult because of the size of the tumor, and its relationship to neurovascular structures and the urinary and intestinal tracts. Among the surgeries for bone tumors, pelvic reconstruction after the resection of bone sarcomas is challenging. Angelini et al. reported that pelvic reconstruction was an independent significant risk factor for infection, and 46% of patients with infection required removal of the reconstruction [1].

Our present study’s results showed that use of implant was associated with an increased risk of postoperative deep infection. In general surgery, biomaterial has been considered to be a risk factor of postoperative deep infection [26]. Previous studies have reported that 9–28% of cases of deep infection occur after endoprosthetic reconstruction [2,4,16,17]. In contrast, reconstruction without an implant is associated with a low infection rate (0.9–1.2%) [19,27,28]. However, infection following biological reconstruction using allograft or autograft is common. Mankin reported that 13% of patients treated with allograft experienced infection [29]. Tsuchiya et al. reported that 11% of patients, who underwent reconstruction using autograft containing tumor treated by liquid nitrogen, had postoperative deep infection [30]. Our study’s findings showed marginal significance for a correlation between biological reconstruction and postoperative deep infection. Thus, the presence of biological reconstruction may influence the incidence of infection.

To decrease postoperative deep infection, preventive care, including drainage and the administration of prophylactic antibiotics, is needed after bone tumor surgeries. Recently, several new techniques, including antibiotic-impregnated cement and an implant with silver coating or iodine coating, have been used to prevent deep infection after orthopaedic surgery [31–34]. More efforts should be made to decrease postoperative deep infection in patients with a high risk of infection.

In conclusion, our present study’s findings showed that a pelvic tumor and the use of an implant are associated with an increased risk of postoperative deep infection. Surgeons will be able to use this information when deciding which operative procedure to use to treat patients with bone tumors.
